# Gene Therapy-Mediated Partial Reprogramming Extends Lifespan and Reverses Age-Related Changes in Aged Mice

**DOI:** 10.1089/cell.2023.0072

**Published:** 2024-02-15

**Authors:** Carolina Cano Macip, Rokib Hasan, Victoria Hoznek, Jihyun Kim, Yuancheng Ryan Lu, Louis E. Metzger, Saumil Sethna, Noah Davidsohn

**Affiliations:** ^1^ Rejuvenate Bio, San Diego, California, USA.; ^2^ Department of Biology, Whitehead Institute for Biomedical Research, Cambridge, Massachusetts, USA.

**Keywords:** AAV, OSK, epigenetic, gene therapy, age reversal

## Abstract

Aging is a complex progression of changes best characterized as the chronic dysregulation of cellular processes leading to deteriorated tissue and organ function. Although aging cannot currently be prevented, its impact on life- and healthspan in the elderly can potentially be minimized by interventions that aim to return these cellular processes to optimal function. Recent studies have demonstrated that partial reprogramming using the Yamanaka factors (or a subset; *OCT4*, *SOX2,* and *KLF4; OSK)* can reverse age-related changes *in vitro* and *in vivo*. However, it is still unknown whether the Yamanaka factors (or a subset) are capable of extending the lifespan of aged wild-type (WT) mice. In this study, we show that systemically delivered adeno-associated viruses, encoding an inducible OSK system, in 124-week-old male mice extend the median remaining lifespan by 109% over WT controls and enhance several health parameters. Importantly, we observed a significant improvement in frailty scores indicating that we were able to improve the healthspan along with increasing the lifespan. Furthermore, in human keratinocytes expressing exogenous OSK, we observed significant epigenetic markers of age reversal, suggesting a potential reregulation of genetic networks to a younger potentially healthier state. Together, these results may have important implications for the development of partial reprogramming interventions to reverse age-associated diseases in the elderly.

## Introduction

The world's population is growing older, with a doubling of the median age from 1900 to 2020, leading to increased societal burden (Partridge et al., 2018). Aging is the strongest risk factor for most common human diseases (Partridge, 2014), hence it is imperative to identify antiaging interventions to delay or even potentially reverse the aging process. Increasing longevity has historically referred to extending the “lifespan” of an organism through various interventions such as public health policies (Merrill, 2014), caloric restriction (de Cabo et al., 2014; López-Otín et al., 2013; Swindell, 2012), or through pharmaceutical interventions (Blagosklonny, 2019; Glossmann and Lutz, 2019).

One potential pitfall of increasing longevity is that it may not necessarily improve quality of life or healthspan. For example, an organism might live longer but still undergo age-related diseases and physiological decline, although over an extended period. In contrast, age reversal involves restoring an organism to a younger state, counteracting the effects of aging at the cellular level, and, consequently, improving both health- and lifespan.

The other pitfall of longevity research is cycle time. For assessment and development of potentially efficacious interventions, it would necessitate waiting for the organism to die. Many groups are working to elucidate biomarkers that are sensitive and correlate reliably with increased lifespan (Horvath, 2013; Horvath and Raj, 2018; Hsu et al., 2020), yet the current gold standard remains “time to death.” This readout works well for short-lived multicellular model organisms such as *Caenorhabditis elegans* (∼3 weeks) (Zhang et al., 2020) and *Drosophila melanogaster* (∼70 days) (Piper and Partridge, 2018). At the mouse level, testing antiaging interventions can take 0.5 to 3 years.

Using a cocktail of transcription factors, *OCT4* (O), *SOX2* (S), *KLF4* (K), and *c-MYC* (M), collectively known as OSKM or Yamanaka factors, seminal studies showed that somatic cells can be reversed to a pluripotent state (Takahashi and Yamanaka, 2006), thereby reversing a long-held paradigm of unidirectional differentiation. By short or cyclic induction of the Yamanaka factors in transgenic mice, investigators have demonstrated age extension in progeroid mice. These transgenic mouse models encoded a polycistronic OSKM cassette driven by a reverse tetracycline transactivator (*rtTA*) (4F mice); cyclic administration of doxycycline led to partial reprogramming without teratoma formation.

This paradigm partially ameliorated aging phenotypes and extended the lifespan in the 4F-progeroid model (Ocampo et al., 2016). Further studies showed that the epigenetic profile assessed by epigenetic methylation clocks (Browder et al., 2022; Chondronasiou et al., 2022; Horvath, 2013; Horvath and Raj, 2018) was rejuvenated by cyclic OSKM induction in several tissues, correlated with their improved function. Another study demonstrated that short induction of OSKM in a myocardial infarction model alleviated myocardial damage and improved cardiac function (Chen et al., 2021).

The translation of these proof-of-concept genetic studies into therapeutic interventions holds promise for the growing aging population but encounters two significant challenges: (1) *c-Myc* (M) in the OSKM cocktail is an oncogene, and its overexpression can lead to the development of tumors and (2) OSKM is too large to be accommodated within existing therapeutic delivery approaches, such as adeno-associated viruses (AAVs).

The aforementioned challenges were effectively addressed by another previous study (Lu et al., 2020), demonstrating the dispensability of c-Myc for rejuvenation, allowing OSK to be packaged into *AAV* as a single polycistron. When packaged by AAV2 capsid and delivered intravenously to the eye, AAV–*OSK* presents the ability to rejuvenate transcriptome and methylome in retinal ganglion cells, leading to axon regeneration and vision restoration in aged and glaucoma mice (Lu et al., 2020) and recently in nonhuman primates of NAION disease (Ksander et al., 2023).

Age-related histone markers were reversed by OSK in the kidney and muscle (Yang et al., 2023), and continued long-term OSK expression through AAV in the eye or liver was ostensibly safe for up to 21 months (Karg et al., 2023; Lu et al., 2020).

However, the critical question of whether partial reprogramming can extend lifespan in wild-type (WT) animals remains unaddressed, highlighting the urgent need for investigation, preferably through a therapeutically feasible method. In support of this endeavor, we independently generated a systemically delivered two-part AAV system with doxycycline-inducible OSK. By cyclic induction of AAV9-mediated OSK expression in 2-year-old WT mice, we observed a remarkable 109% increase in median remaining life with improved health condition relative to doxycycline-treated control mice. Moreover, we showed that such treatments lead to profound age reversal in the heart and liver tissues, as well as human keratinocytes, as assessed by DNA methylation clocks.

## Materials and Methods

### Vector and AAV generation

Constructs containing tetracycline-responsive element version 3 (TRE3) promoter driving the expression of human *OCT3/4*, *SOX2*, and *KLF4* from a polycistronic transcript (TRE3-*OSK*) and second construct encoding *rtTA* version 4 driven by *hEf1a* promoter (hEf1a-*rtTA4*) were generated by Genscript (Piscataway, NJ) as reported previously (Lu et al., 2020). The constructs were packaged in AAV9 capsid to generate AAV9.TRE3-*OSK*-SV40pA (1.556 E13 vg/mL) and AAV9-hEf1a-*rtTA4*-Sv40pA (1.88 E13 vg/mL) by SignaGen (Fredrick, MD).

### Mouse studies and frailty scores

Mouse experiments were performed at Jax laboratories (AUS protocol #19063). Male C57BL6/J (JAX Stock# 000664) mice aged to 124 weeks were injected with the two viruses described above: each 1E12 vg/mouse (in 100 μL volume) through retro-orbital route. Control mice were injected with 100 μL formulation buffer (phosphate buffered saline [PBS]). Doxycycline induction was performed 1 week on/1 week off for the duration of the study, by providing 2 mg/mL final concentration of doxycycline in drinking water, same as prior AAV–OSK study (Lu et al., 2020).

Control mice received doxycycline in water at the same concentration as the vector-injected mice. The euthanasia criteria were as follows: a rapid or sustained deterioration in health status resulting in a body condition score of ≤2; tumors or other masses that become ulcerated or interfere with the ability of the animal to eat, drink, or ambulate; any prolapsed organs that cannot be reduced and/or become ulcerated and/or necrotic; any other condition that interferes with ability to reach or consume adequate amounts of food or water.

Mice were individually weighed and assessed across 28 different variables including physical, physiological, and innate reflex conditions, including simple sensorial and motor tests, body temperature, and overall body condition assessment. A frailty index (FI) score (Heinze-Milne et al., 2019) is calculated per mouse by adding all individual scores (excluding body temperature and weight) together detailed in Supplementary Table S2.

### DNA extraction from tissues and DNA methylation age measurement

Mice that were healthy and euthanized at the end of the study were selected for methylation studies. Tissue from the liver and the heart was extracted using the DNeasy Blood and Tissue Kit (Quiagen), following the manufacturer's protocol. Methylation analysis on the above extracted DNA was performed by the Clock Foundation (Torrence, CA). Lifespan Uber Correlation (LUC) clock algorithm and analysis has been described previously (Browder et al., 2022; Haghani et al., 2022).

### RNA extractions and quantitative polymerase chain reaction (qPCR)

RNA was extracted using the Qiazol (Qiagen) and chloroform phase separation method. cDNA was synthesized using the PrimeScript 1st strand cDNA synthesis kit (Takara Bio). Reaction was performed with the PowerUp SYBR Green Master Mix (Applied Biosystems) and following their recommended protocol.

Primers used for qPCR

**Table:** 

Primer pairs	Forward primer	Reverse primer
Oct 4	GGCTTCAGACTTCGCCTTCT	TGGAAGCTTAGCCAGGTTCG
Sox 2	TTTGTCCGAGACCGAGAAGC	CTCCGGGAAGCGTGTACTTA
KLF 4	GCACACCTGCGAACTCACAC	CCGTCCCAGTCACAGTGGTAA
GAPDH	GGCAAATTCAACGGCACAGT	GTCTCGCTCCTGGAAGATGG

### Lentivirus production and keratinocyte transduction

Plasmids encoding polycistronic OSK driven by human *EF1ɑ* promoter, *PsPax2*, and *PmD2.G* were cotransfected into HEK293T cells with PEI and Opti-MEM (Gibco). The next day, the medium was replaced with harvest medium containing DMEM, 15% FBS, and 1% PenStrep (Gibco). Supernatant was collected on days 3 and 4, filtered through a 0.45 polyethersulfone membrane, and 1 × volume of Lenti-X concentrator (Takara Bio) was combined with 3 × volumes of clarified viral medium and stored at 4°C overnight. Viral medium was spun at 1500 *g* for 45 minutes and pellet was resuspended in DMEM. Virus was titered using Lenti-X GoStix Plus (Takara Bio). Lentivirus encoding GFP was purchased from VectorBuilder (Chicago, IL).

Lentivirus containing medium was added dropwise to HEK001 (ATCC CRL-2404) passage 110 containing 8 μg/mL of polybrene (Millipore-Sigma) at 2 different MOIs: 0.5 and 1.0. Puromycin at 1 ng/μL (Millipore-Sigma) was added on day 2 for selection. Surviving cells were expanded and maintained with puromycin, changing medium every 3 days and splitting as necessary. On day 23 after selection, cells were fully recovered from puromycin selection and thus collected for immunoblot and methylation analysis.

### Immunoblot analysis

Protein from cells described above was extracted on ice using Lysis Buffer [Cell Signaling Technology (CST)] with 1 mM phenylmethylsulfonyl fluoride (PMSF) and protease inhibitor cocktail. Cell lysis mixture was spun for 10 minutes at 14,000 g/4°C, supernatant was collected, and protein was quantified using Pierce Rapid Gold BCA Protein Assay Kit (Thermo Fisher). Equal amounts of protein were loaded in a 4%–15% polyacrylamide gel (Thermo Fisher) and transferred to polyvinylidene difluoride (PVDF) membranes and blocked with 5% dry milk in Tris-buffered saline with 0.1% Tween^®^ 20 detergent (TBST) for 1 hour at room temperature.

Membranes were incubated overnight at 4°C with primary antibodies. The following day, membranes were incubated in a secondary antibody conjugated to horse radish peroxidase (HRP) for 1 hour at room temperature and developed with ECL Prime Western Blotting Detection Reagent (Civita Life Sciences).

Antibodies used for immunoblot

**Table:** 

Name	Cat. No.	Manufacturer	Dilution	Host species
Oct4	Ab181557	Abcam	1:1000	Rabbit
Sox2	Ab92494	Abcam	1:1000	Rabbit
Klf4	PA5–20897	Thermo Fisher	1:1000	Rabbit
GAPDH-AF488	MAB374-AF488	Millipore Sigma	1:1000	Mouse
Rabbit IgG-HRP	Ab6721	Abcam	1:2500	Goat

## Results

Transgenic mouse models lack suitability for translating therapeutic strategies to humans for age reversal. Therefore, we employed an AAV system for the systemic delivery of OSK. In addition, as age reversal therapeutics are not intended for young humans, we selected extremely old mice (124 weeks) as a model system to enhance translatability. WT C57BL6/J mice have a median lifespan of ∼129 weeks (Yuan et al., 2012), equivalent to ∼80 years in humans (Ackert-Bicknell et al., 2015).

**FIG. 1.**
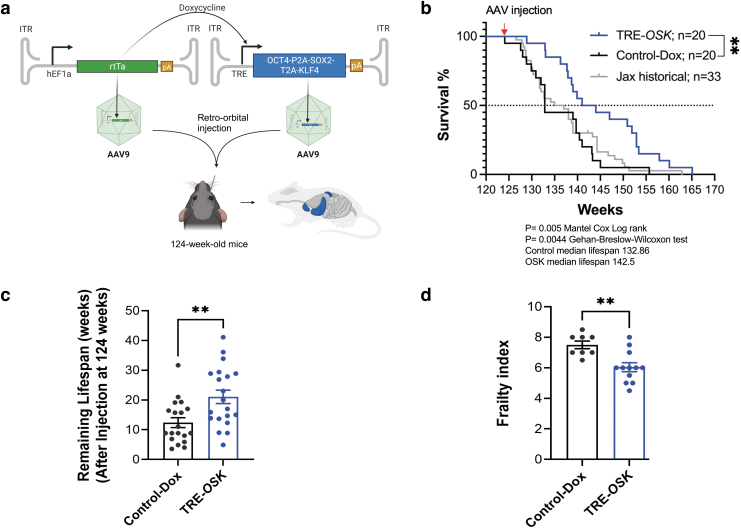
Partial reprogramming with TRE-*OSK* leads to increased lifespan and improved frailty scores in very old mice. **(a)** Schematic of the constructs, virus, and injection route used in the study. **(b)** Kaplan–Meier curves for 124-week WT mice injected with AAV9.TRE-*OSK* and AAV9.hEF1ɑ-*rtTA4* (both 1E12 vg/animal) through the retro-orbital route, and induced with 1 week on/off doxycycline paradigm (TRE-*OSK*) showed median lifespan extension of remaining life by 109% compared with either doxycycline-treated control animals (Control-Dox) or to historical published Jax data for Bl6/J mice (Jax historical). Red arrow at top indicates AAV injections. Mantel Cox Log rank test, **p < 0.05. **(c)** Graph shows remaining lifespan of individual mice (after injections at week 124) for data shown in **(b)**. Two-tailed unpaired *t*-test; **p < 0.05. **(d)** FI, the compound score of 28 different health parameters (range 0–1 in 0.5 increments), showed significant reduction in FI for TRE-*OSK* mice at 142 weeks of age (18 weeks after injections) as compared with Control-Dox mice. Student's unpaired *t*-test, **p < 0.05. AAV, adeno-associated virus; FI, frailty index; WT, wild-type.

We drove inducible OSK expression in 124-week mice (∼77-year-old equivalent human age) using a two-part AAV system, where one vector carried a constitutively expressed *rtTA* and the other vector contained a polycistronic OSK expression cassette driven by doxycycline responsive TRE promoter (Lu et al., 2020) (Fig. 1a). A more tightly regulated version of *rtTA* (*rtTA4*) was employed due to its superior performance in minimizing leaky liver expression in the absence of doxycycline and faster on/off switching compared with the traditional *rtTA3* (Sinclair et al., 2021).

We selected AAV9 capsid and *EF1a* promoter to ensure maximal distribution to most tissues (Inagaki et al., 2006 and Supplementary Table S1). We injected 124-week-old WT C57BL6/J mice retro-orbitally with 100 μL containing either PBS (formulation buffer) or 1E12 vg of each vector for a total dose of ∼6E13 vg/kg. We initiated the doxycycline induction for both the control and AAV–*OSK* administered groups the day after injections and alternated weekly on/off cycles for the remainder of the animals' lives (details given in Materials and Methods section**)**.

Doxycycline-treated control mice had a median lifespan of ∼133 weeks, whereas the TRE-*OSK* mice had a median lifespan of 142.5 weeks (Fig. 1b, c and Supplementary Fig. S1): a remarkable 109% extension in median remaining life in response to OSK expression (control mice had 8.86 weeks of life remaining vs. 18.5 weeks for TRE-*OSK* mice). We further compared the control doxycycline-treated mice with the historical published data for BL6/J mice (Yuan et al., 2012) and available through the mouse phenome database (https://phenome.jax.org/projects/Yuan2); we found no significant differences in median survival, suggesting that doxycycline alone had no adverse nor advantageous effects (Fig. 1b).

In addition, a previous report has demonstrated that control AAV9 expressing GFP at the same dose we used (2e12vg/mouse) does not alter median lifespan when administered to 2-year-old mice (Bernardes de Jesus et al., 2012).

Aging is associated with an increased susceptibility to adverse health outcomes that can be captured by clinicians using a FI, where people are scored based on a subset of age-related health deficits. High compound scores reflect a frail state and increased susceptibility to poor health outcomes (Searle et al., 2008). A similar index can be used in mice to assess aging and effects of aging interventions (Heinze-Milne et al., 2019). We observed a significant reduction in the FI from 7.5 points for doxycycline-treated control mice to 6 points for TRE-*OSK* mice (Fig. 1d, *p* = 0.0027), suggesting that increased lifespan correlated with overall better health of the animals.

Molecular measures of cellular and tissue health have been developed based on methylation patterns of genomic DNA. “Epigenetic age,” a well characterized and established aging biomarker, can be calculated using these methylation patterns. Such epigenetic clock biomarkers decouple chronological age (Bell et al., 2019) from the functional state of the cells or tissues, while correlating better to aging, disease state(s), and health outcomes (Bell et al., 2019; Durso et al., 2017; Fransquet et al., 2019; Xiao et al., 2021).

**FIG. 2.**
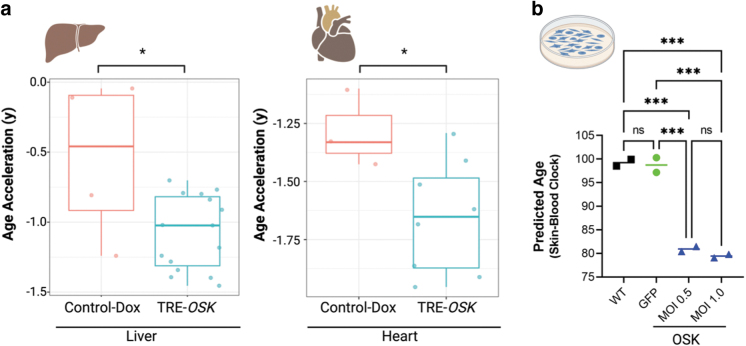
Partial reprogramming with TRE-*OSK* leads to age reversal as assessed by DNA methylation age. **(a)** Measurement of DNA methylation age acceleration in mouse liver (*left panel*) and heart (*right panel*) from control doxycycline-treated mice (Control-Dox) or TRE-*OSK* mice using LUC epigenetic clocks trained on the indicated tissues. Age acceleration is the difference between clock age and chronological age (Supplementary Table S3) *p < 0.05, *p* = 0.0139 for liver and *p* = 0.0414 for heart by unpaired *t*-test. **(b)** Human keratinocytes isolated from the scalp of a 65-year-old male patient transduced with lentivirus at two different MOIs (0.5 and 1.0) expressing OSK showed epigenetic age reversal as compared with control GFP transduced or nontransduced (WT) cells. *n* = 2 technical repeats for each group. One-way ANOVA with Holm–Šídák's multiple comparisons test. ***p < 0.001. LUC, Lifespan Uber Correlation; ns, not significant.

Matching with previous reports of AAV9 tissue tropism (Supplementary Table S1), we observed that high OSK expression in the liver and heart, but contrary to AAV9, failed to see high expression levels in the brain of mice that received AAV9-EF1a-*rtTA4* and TRE-*OSK* (Supplementary Fig. S2). The lack of brain expression of OSK is likely to low cotransduction of dual AAVs and lower DOX penetration. Therefore, we isolated DNA from heart and liver tissue from control and TRE-*OSK*-treated mice at time of death and measured the epigenetic age with the LUC clock, which correlates age-related CpGs with maximum lifespan (Browder et al., 2022; Haghani et al., 2022). Both liver and heart from the OSK treatment group have significantly reduced epigenetic age compared with control (Fig. 2a).

To assess the rejuvenation effects of OSK overexpression in human cells, we expressed OSK in HEK001 keratinocytes isolated from the scalp of a 65-year-old male patient, even though this cell line has been immortalized to prevent replicative senescence, several studies by the Steven Horvath and Ken Raj group show that the increase of DNAm age is not prevented by immortalization (Kabacik et al., 2022; Kabacik et al., 2018; Lu et al., 2018). We confirmed, by immunoblot, the exogenous expression of OSK in these keratinocytes transduced with lentivirus (Supplementary Fig. 3a–c).

Next, we found significant epigenetic age reversal in keratinocytes treated with OSK as compared with either untransduced or GFP transduced cells (Fig. 2b). Taken together, our mouse and keratinocytes data suggest that AAV-mediated gene therapy delivering OSK increases lifespan in mice with improved health parameters and reverses biomarkers of aging in mouse and human cells.

The necessity for cotransduction of both AAVs (EF1a-*rtTA4* and TRE-*OSK*) into the same cell could potentially limit tissue distribution, hindering the observation of more extensive whole-body rejuvenation and a greater extension of lifespan. Considering the safety of continued OSK expression through AAV in the eye or liver for up to 21 months (Karg et al., 2023; Lu et al., 2020), coupled with sustained vision improvement surpassing that of a cyclic OSK regimen (Karg et al., 2023), we engineered a single noninducible AAV vector (pAAV-CMV-*OSK*) to explore its potential to reach tissues in aged mice with reduced AAV titers.

**FIG. 3.**
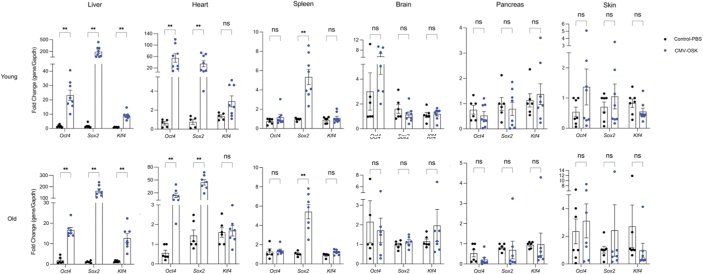
AAV9-CMV-*OSK* expression in tissues of young and aged mice. qPCR of *Oct4*, *Sox2*, and *Klf4* from 8-week-old (Young) and 82-week-old (Old) mice retro-orbitally injected with the AAV9 1.7e11 vg/mouse and uninfected controls, six different tissues were collected 12 weeks after injection. Level of each gene is normalized to that of controls receiving PBS. Multiple Mann–Whitney test with Holm–Šídák's multiple comparisons. **p < 0.05. PBS, phosphate buffered saline.

Remarkably, using 1/10th of the AAV at 1.7e11 vg/mouse, we observed robust OSK expression in the liver, heart, and spleen of both 8-week-old and 82-week-old mice (Fig. 3). This encourages further investigation into the noninducible single OSK AAV's capacity to rejuvenate tissues and its impact on lifespan in future studies.

## Discussion

In modern societies, aging is the highest risk factor associated with most diseases and mortality (Partridge, 2014). The goals of regenerative medicine are to improve tissue and organ function and to correct disease states. Cellular rejuvenation through partial reprogramming has been shown to be a promising avenue to achieve the goals of regenerative medicine, as it targets the epigenetic information loss during aging and injury (Lu et al., 2023; Yang et al., 2023). Here we show that in human cells, exogenous expression of an OSK leads to profound age reversal as observed by the restoration of genomic methylation patterns to those that are characteristic of younger cells, a validated hallmark of chronological age reversal (Bell et al., 2019; Haghani et al., 2022; Xiao et al., 2021).

To our knowledge, we have shown for the first time an extension of remaining median lifespan in extremely old WT C57BL6/J mice concomitantly with improved health outcomes as a consequence of a systemic AAV-based partial reprogramming therapy. Experiments to assess the epigenetic programming hallmarks in specific tissues, along with thorough analysis of the RNA profiles at single cell level (Roux et al., 2022), will be required to make broader conclusions as to which pathways are reprogrammed to a more youthful state.

Teratoma formation has been observed in partially reprogrammed animals, particularly when *c-Myc* is used in the partial rejuvenation cocktail (Abad et al., 2013; Ocampo et al., 2016; Ohnishi et al., 2014; Senís et al., 2018). Although poorly invasive and poorly metastatic, teratoma formation is unlikely to be accepted by the FDA, hence tight control of the partial rejuvenation factors will be a key attribute for safe and efficacious rejuvenation therapies. We did not notice any gross teratoma formation when we processed the tissues from animals receiving AAV9-EF1a-*rtTA4*; TRE-*OSK* or AAV9-CMV-*OSK*, or during the frailty score measurement (Supplementary Table S2).

These observations, along with recent advances in vector development and optimization, tissue-specific promoters, and inducible systems (Domenger and Grimm, 2019; Li and Samulski, 2020), engender cautious optimism that a partial rejuvenation therapy can be safely delivered in humans. Prudent and thorough monitoring studies in large animals will be required to assess the safety and efficacy of partial rejuvenation studies.

We assessed whether *in vivo* partial cellular rejuvenation is sufficient to extend lifespan and healthspan in a relevantly old population and to remove a major barrier to the systemic delivery of three Yamanaka factors within a single vector. Investigators have hitherto shown transduction of specific organs with combinations of OSK or OSKM, but with each encapsulated in a separate vector (Senís et al., 2018). For therapeutic development in humans, having three separate vectors significantly increases the complexity of manufacturing, drug product specifications, and administration protocols for clinical development.

Based on our novel proof-of-concept studies in an extremely aged mouse population (equivalent to >80 years of age in humans) and previous studies in younger mice (Browder et al., 2022; Lu et al., 2020; Ocampo et al., 2016), we envision therapeutic rejuvenation in aged humans, first in a specific age-related disease setting and later for therapeutic healthspan and lifespan extension.

### Limitation of the study

Although we showed a lifespan extension with AAV–*OSK* compared with DOX-treated control mice and JAX historical mice lifespan, it would be ideal to have an additional control group of AAV scramble or AAV-GFP to rule out any potential effect of AAV. That said, previous report has already demonstrated that control AAV (AAV9-*GFP*) infection (at the same dose used in this study) in 2-year-old mice does not alter median lifespan at all (Bernardes de Jesus et al., 2012). Due to a limited availability of aged female mice, we focused our investigation solely on male subjects.

We examined AAV expression in limited tissues from mice in the lifespan study, from the combined consequences of a cyclic induction protocol and allowing the mice to reach a humane endpoint resulted in most mice being found dead instead of euthanized. The cyclic induction protocol meant that at any given time a mouse died, only half the mice are expressing OSK and even less than that are maximally expressing OSK, as maximal expression would only be achieved on day 7 of Dox induction. However, we were still able to observe some expression in liver and heart, and our single constitutive AAV–*OSK* vector tissue distribution data suggest additional spleen expression (Fig. 3).

Other data from published literature on AAV9 tissue tropism suggest that there are potentially more tissues with OSK transduction, however, the need for coinfection and doxycycline bioavailability appears to further limit which tissues can overexpress OSK (Supplementary Table S1). A more definitive examination should involve tissue distribution examination with luciferase, replacing OSK (Liao et al., 2017; Lu et al., 2020). Future studies can investigate this aspect to help design vectors that can more systemically reverse tissue age.

## Supplementary Material

Supplemental data

Supplemental data

Supplemental data

Supplemental data

Supplemental data

## References

[B1] Abad M, Mosteiro L, Pantoja C, et al. Reprogramming in vivo produces teratomas and iPS cells with totipotency features. Nature 2013;502(7471):340–345; doi: 10.1038/nature12586 24025773

[B2] Ackert-Bicknell CL, Anderson LC, Sheehan S, et al. Aging research using mouse models. Curr Protoc Mouse Biol 2015;5(2):95–133; doi: 10.1002/9780470942390.mo140195 26069080 PMC4590775

[B3] Bell CG, Lowe R, Adams PD, et al. DNA methylation aging clocks: Challenges and recommendations. Genome Biol 2019;20(1):249; doi: 10.1186/s13059-019-1824-y 31767039 PMC6876109

[B4] Bernardes de Jesus B, Vera E, Schneeberger K, et al. Telomerase gene therapy in adult and old mice delays aging and increases longevity without increasing cancer. EMBO Mol Med 2012;4(8):691–704; doi: 10.1002/emmm.201200245 22585399 PMC3494070

[B5] Blagosklonny MV. Rapamycin for longevity: Opinion article. Aging (Albany NY) 2019;11(19):8048–8067; doi: 10.18632/aging.102355 31586989 PMC6814615

[B6] Browder KC, Reddy P, Yamamoto M, et al. In vivo partial reprogramming alters age-associated molecular changes during physiological aging in mice. Nature Aging 2022;2(3):243–253; doi: 10.1038/s43587-022-00183-2 37118377

[B7] Chen Y, Lüttmann FF, Schoger E, et al. Reversible reprogramming of cardiomyocytes to a fetal state drives heart regeneration in mice. Science 2021;373(6562):1537–1540; doi: 10.1126/science.abg5159 34554778

[B8] Chondronasiou D, Gill D, Mosteiro L, et al. Multi-omic rejuvenation of naturally aged tissues by a single cycle of transient reprogramming. Aging Cell 2022;21(3):e13578; doi: 10.1111/acel.13578 35235716 PMC8920440

[B9] de Cabo R, Carmona-Gutierrez D, Bernier M, et al. The search for antiaging interventions: From elixirs to fasting regimens. Cell 2014;157(7):1515–1526; doi: 10.1016/j.cell.2014.05.031 24949965 PMC4254402

[B10] Domenger C, Grimm D. Next-generation AAV vectors-do not judge a virus (only) by its cover. Hum Mol Genet 2019;28(R1):R3–r14; doi: 10.1093/hmg/ddz148 31261383

[B11] Durso DF, Bacalini MG, Sala C, et al. Acceleration of leukocytes' epigenetic age as an early tumor and sex-specific marker of breast and colorectal cancer. Oncotarget 2017;8(14):23237–23245; doi: 10.18632/oncotarget.15573 28423572 PMC5410300

[B12] Fransquet PD, Wrigglesworth J, Woods RL, et al. The epigenetic clock as a predictor of disease and mortality risk: A systematic review and meta-analysis. Clin Epigenetics 2019;11(1):62; doi: 10.1186/s13148-019-0656-7 30975202 PMC6458841

[B13] Glossmann HH, Lutz OMD. Metformin and aging: A review. Gerontology 2019;65(6):581–590; doi: 10.1159/000502257 31522175

[B14] Hagani A, Wang N, Lu AT, et al. Divergent age-related methylation patterns in long and short-lived mammals. bioRxiv 2022;2022:476530; doi: 10.1101/2022.01.16.476530

[B15] Horvath S. DNA methylation age of human tissues and cell types. Genome Biol 2013;14(10):R115; doi: 10.1186/gb-2013-14-10-r115 24138928 PMC4015143

[B16] Horvath S, Raj K. DNA methylation-based biomarkers and the epigenetic clock theory of ageing. Nat Rev Genet 2018;19(6):371–384; doi: 10.1038/s41576-018-0004-3 29643443

[B17] Hsu YH, Astley CM, Cole JB, et al. Integrating untargeted metabolomics, genetically informed causal inference, and pathway enrichment to define the obesity metabolome. Int J Obes (Lond) 2020;44(7):1596–1606; doi: 10.1038/s41366-020-0603-x 32467615 PMC7332400

[B18] Inagaki K, Fuess S, Storm TA, et al. Robust systemic transduction with AAV9 vectors in mice: Efficient global cardiac gene transfer superior to that of AAV8. Mol Ther 2006;14(1):45–53; doi: 10.1016/j.ymthe.2006.03.014 16713360 PMC1564441

[B19] Kabacik S, Horvath S, Cohen H, et al. Epigenetic ageing is distinct from senescence-mediated ageing and is not prevented by telomerase expression. Aging (Albany, NY) 2018;10(10):2800–2815; doi: 10.18632/aging.101588 30332397 PMC6224244

[B20] Kabacik S, Lowe D, Fransen L, et al. The relationship between epigenetic age and the hallmarks of aging in human cells. Nat Aging 2022;2(6):484–493; doi: 10.1038/s43587-022-00220-0 37034474 PMC10077971

[B21] Karg MM, Lu YR, Refaian N, et al. Sustained vision recovery by OSK gene therapy in a mouse model of glaucoma. Cell Reprog 2023;25(6):288–299; doi: 10.1089/cell.2023.0074 PMC10739681 38060815

[B22] Ksander B, Shah M, Krasniqi D, et al. Epigenetic reprogramming-A novel gene therapy that restores vision loss in a nonhuman primate model of NAION. Invest Ophthalmol Vis Sci 2023;64(8):474–474.

[B23] Li C, Samulski RJ. Engineering adeno-associated virus vectors for gene therapy. Nat Rev Genet 2020;21(4):255–272; doi: 10.1038/s41576-019-0205-4 32042148

[B24] Liao HK, Hatanaka F, Araoka T, et al. *In vivo* target gene activation via CRISPR/Cas9-mediated trans-epigenetic modulation. Cell 2017;171(7):1495–1507 e1415; doi: 10.1016/j.cell.2017.10.025 29224783 PMC5732045

[B25] López-Otín C, Blasco MA, Partridge L, et al. The hallmarks of aging. Cell 2013;153(6):1194–1217; doi: 10.1016/j.cell.2013.05.039 23746838 PMC3836174

[B26] Lu AT, Xue L, Salfati EL, et al. GWAS of epigenetic aging rates in blood reveals a critical role for TERT. Nat Commun 2018;9(1):387; doi: 10.1038/s41467-017-02697-5 29374233 PMC5786029

[B27] Lu Y, Brommer B, Tian X, et al. Reprogramming to recover youthful epigenetic information and restore vision. Nature 2020;588(7836):124–129; doi: 10.1038/s41586-020-2975-4 33268865 PMC7752134

[B28] Lu YR, Tian X, Sinclair DA. The information theory of aging. Nat Aging 2023;3(12):1486–1499; doi: 10.1038/s43587-023-00527-6 38102202

[B29] Merrill GBLRM. The contribution of public health and improved social conditions to increased life expectancy: An analysis of public awareness. J Commun Med Health Educ 2014;04:1000311; doi: 10.4172/2161-0711.1000311

[B30] Ocampo A, Reddy P, Martinez-Redondo P, et al. In vivo amelioration of age-associated hallmarks by partial reprogramming. Cell 2016;167(7):1719–1733.e1712; doi: 10.1016/j.cell.2016.11.052 27984723 PMC5679279

[B31] Ohnishi K, Semi K, Yamamoto T, et al. Premature termination of reprogramming in vivo leads to cancer development through altered epigenetic regulation. Cell 2014;156(4):663–677; doi: 10.1016/j.cell.2014.01.005 24529372

[B32] Partridge L. Intervening in ageing to prevent the diseases of ageing. Trends Endocrinol Metab 2014;25(11):555–557; doi: 10.1016/j.tem.2014.08.003 25175302

[B33] Partridge L, Deelen J, Slagboom PE. Facing up to the global challenges of ageing. Nature 2018;561(7721):45–56; doi: 10.1038/s41586-018-0457-8 30185958

[B34] Piper MDW, Partridge L. Drosophila as a model for ageing. Biochim Biophys Acta Mol Basis Dis 2018;1864(9 Pt A):2707–2717; doi: 10.1016/j.bbadis.2017.09.016 28964875

[B35] Roux AE, Zhang C, Paw J, et al. Diverse partial reprogramming strategies restore youthful gene expression and transiently suppress cell identity. Cell Syst 2022;13(7):574–587 e511; doi: 10.1016/j.cels.2022.05.002 35690067

[B36] Searle SD, Mitnitski A, Gahbauer EA, et al. A standard procedure for creating a frailty index. BMC Geriatr 2008;8:24; doi: 10.1186/1471-2318-8-24 18826625 PMC2573877

[B37] Senís E, Mosteiro L, Wilkening S, et al. AAV vector-mediated in vivo reprogramming into pluripotency. Nat Commun 2018;9(1):2651; doi: 10.1038/s41467-018-05059-x 29985406 PMC6037684

[B38] Sinclair DA, Lu Y, Davidsohn NJ. Mutant Reverse Tetracycline Transactivators for Expression of Genes. WO2020069339A1 2021.

[B39] Swindell WR. Dietary restriction in rats and mice: A meta-analysis and review of the evidence for genotype-dependent effects on lifespan. Ageing Res Rev 2012;11(2):254–270; doi: 10.1016/j.arr.2011.12.006 22210149 PMC3299887

[B40] Takahashi K, Yamanaka S. Induction of pluripotent stem cells from mouse embryonic and adult fibroblast cultures by defined factors. Cell 2006;126(4):663–676; doi: 10.1016/j.cell.2006.07.024 16904174

[B41] Xiao C, Beitler JJ, Peng G, et al. Epigenetic age acceleration, fatigue, and inflammation in patients undergoing radiation therapy for head and neck cancer: A longitudinal study. Cancer 2021;127(18):3361–3371; doi: 10.1002/cncr.33641 34027995

[B42] Yang JH, Hayano M, Griffin PT, et al. Loss of epigenetic information as a cause of mammalian aging. Cell 2023;186(2):305–326 e327; doi: 10.1016/j.cell.2022.12.027 36638792 PMC10166133

[B43] Yuan R, Meng Q, Nautiyal J, et al. Genetic coregulation of age of female sexual maturation and lifespan through circulating IGF1 among inbred mouse strains. Proc Natl Acad Sci U S A 2012;109(21):8224–8229; doi: 10.1073/pnas.1121113109 22566614 PMC3361401

[B44] Zhang S, Li F, Zhou T, et al. *Caenorhabditis elegans* as a useful model for studying aging mutations. Front Endocrinol (Lausanne) 2020;11:554994; doi: 10.3389/fendo.2020.554994 33123086 PMC7570440

