# Supplementary material for: Gene Therapy-Mediated Partial Reprogramming Extends Lifespan and Reverses Age-Related Changes in Aged Mice
Source: Cell Reprogram. 2024 Feb 15;26(1):24–32. doi: 10.1089/cell.2023.0072 (PMC10909732; doi:10.1089/cell.2023.0072)
Supplement: Supplemental data [file Supp_TableS1.pdf]

Supplemental Table 1

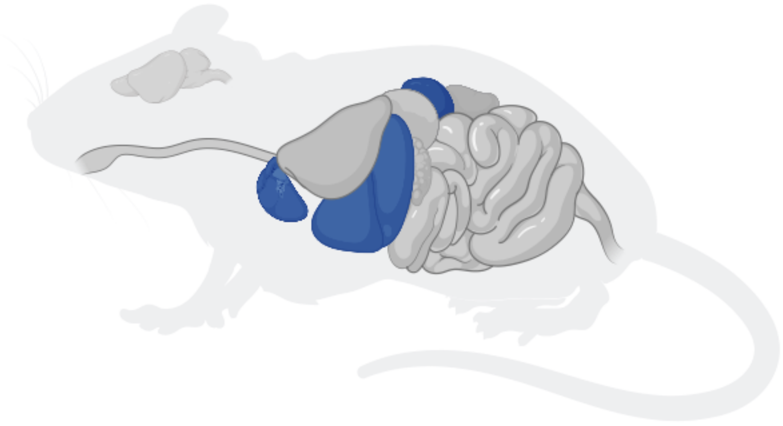

Tissues colored blue exhibit high-confidence expression of OSK.

Tissues expressing GOI by AAV9 with relevant design	Promoter used	Paper
Liver	EF1a	Chandler et al, Hum Mol Genet, 2017<br>Inagaki et al, Mol Therapy, 2006
	UBC-rtTA4;TRE-OSK	Lu et al, Nature, 2020
	EF1a-rtTA4; TRE-OSK	This paper
Heart	EF1a	Inagaki et al, 2006
	EF1a-rtTA4; TRE-OSK	This paper
Spleen	CMV-OSK	This paper
Pancreas	UBC-rtTA4; TRE-OSK	Lu et al, Nature, 2020
Brain*	EF1a	Inagaki et al, 2006<br>Chandler et al, 2017, Hum Mol Genet
	EF1a-rtTA4; TRE-OSK (*not expressed due to the low co-transduction efficiency to the brain)	This paper
