# Supplementary material for: Gene Therapy-Mediated Partial Reprogramming Extends Lifespan and Reverses Age-Related Changes in Aged Mice
Source: Cell Reprogram. 2024 Feb 15;26(1):24–32. doi: 10.1089/cell.2023.0072 (PMC10909732; doi:10.1089/cell.2023.0072)
Supplement: Supplemental data [file Supp_FigS1.docx]

**
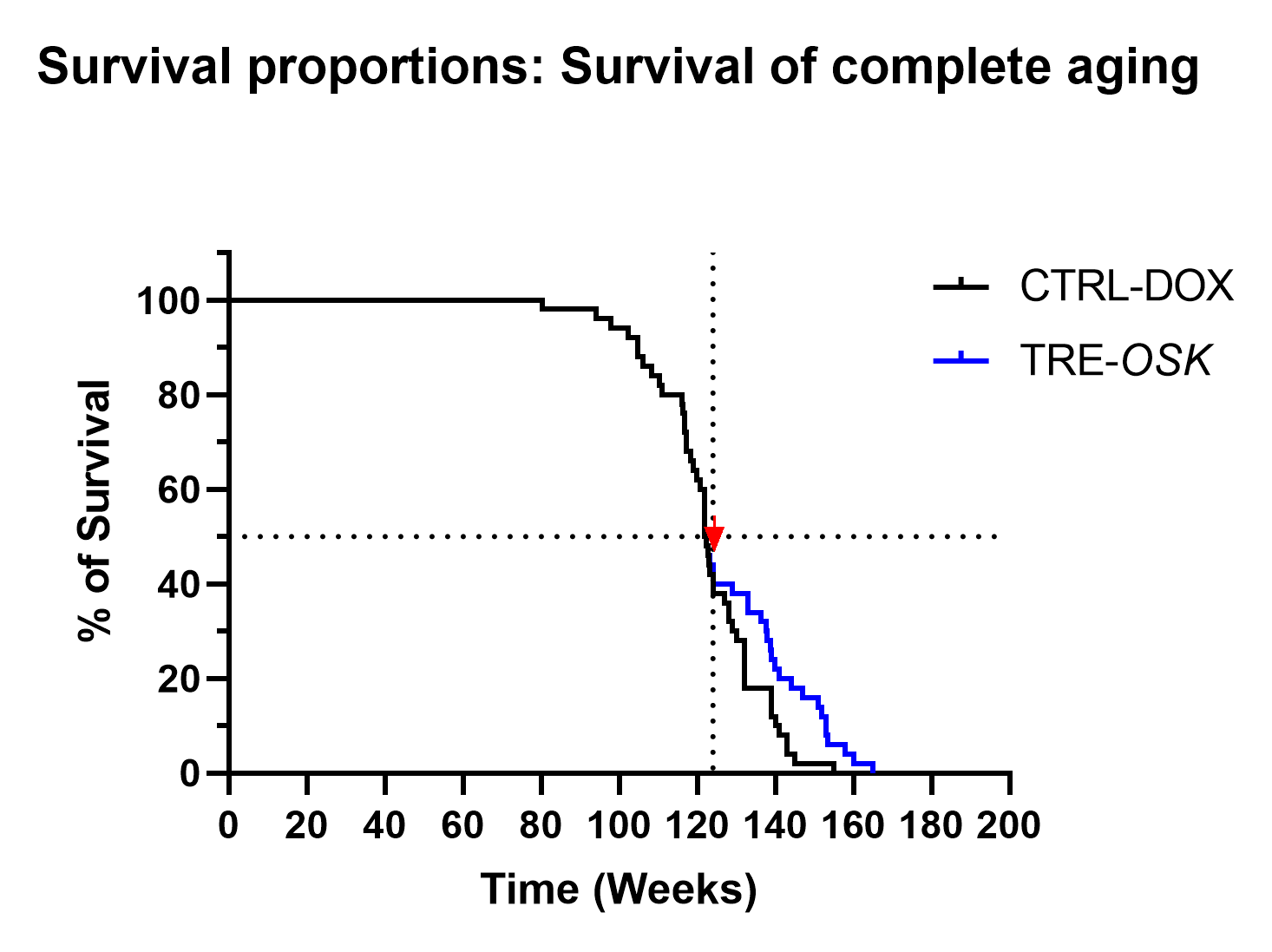
**

**Supplementary Fig. 1: Overall survival proportion curves for control mice and TRE-*OSK* mice over the entire lifespan.**

Survival proportions for all mice purchased for this study during showing the entire time course for data shown in Fig. 1, pre and post injection. Red arrow indicates AAV injections.
