# Supplementary material for: Gene Therapy-Mediated Partial Reprogramming Extends Lifespan and Reverses Age-Related Changes in Aged Mice
Source: Cell Reprogram. 2024 Feb 15;26(1):24–32. doi: 10.1089/cell.2023.0072 (PMC10909732; doi:10.1089/cell.2023.0072)
Supplement: Supplemental data [file Supp_FigS2.docx]

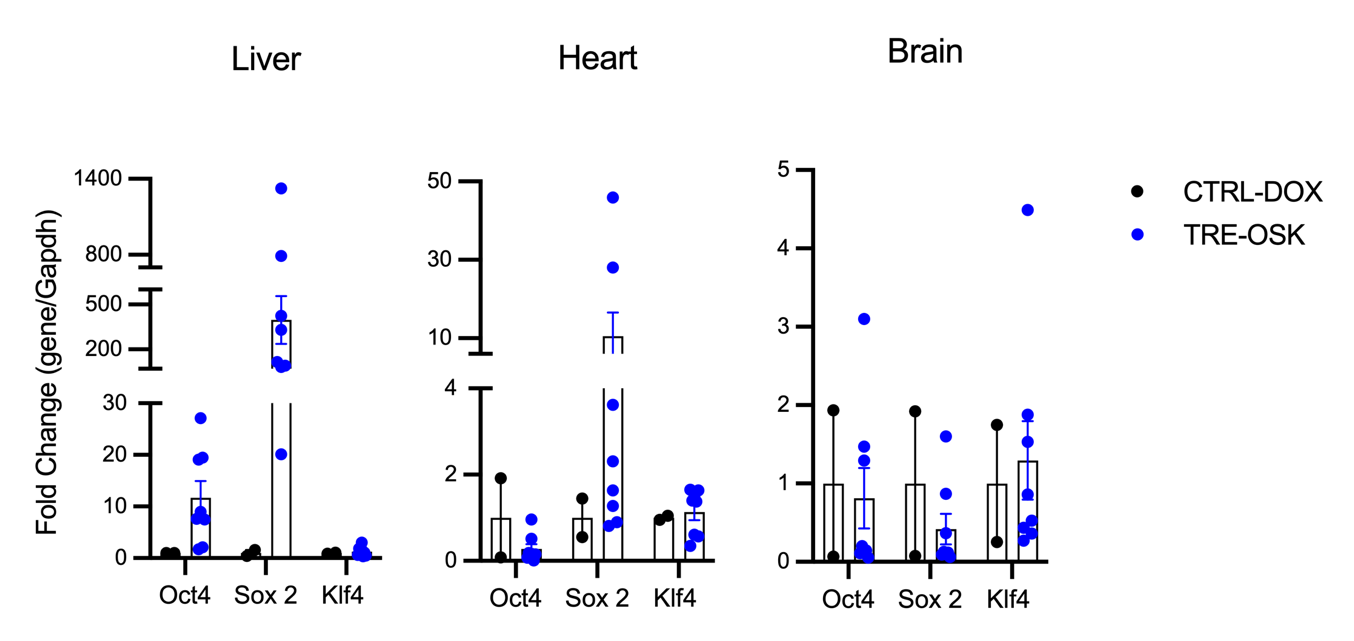


Supplementary Fig. 2: Expression of OSK in the tissues collected from animals died at various time points during the cyclic DOX lifespan experiment. Level of each gene is normalized to controls groups receiving DOX.
