# Supplementary material for: Gene Therapy-Mediated Partial Reprogramming Extends Lifespan and Reverses Age-Related Changes in Aged Mice
Source: Cell Reprogram. 2024 Feb 15;26(1):24–32. doi: 10.1089/cell.2023.0072 (PMC10909732; doi:10.1089/cell.2023.0072)
Supplement: Supplemental data [file Supp_FigS3.docx]

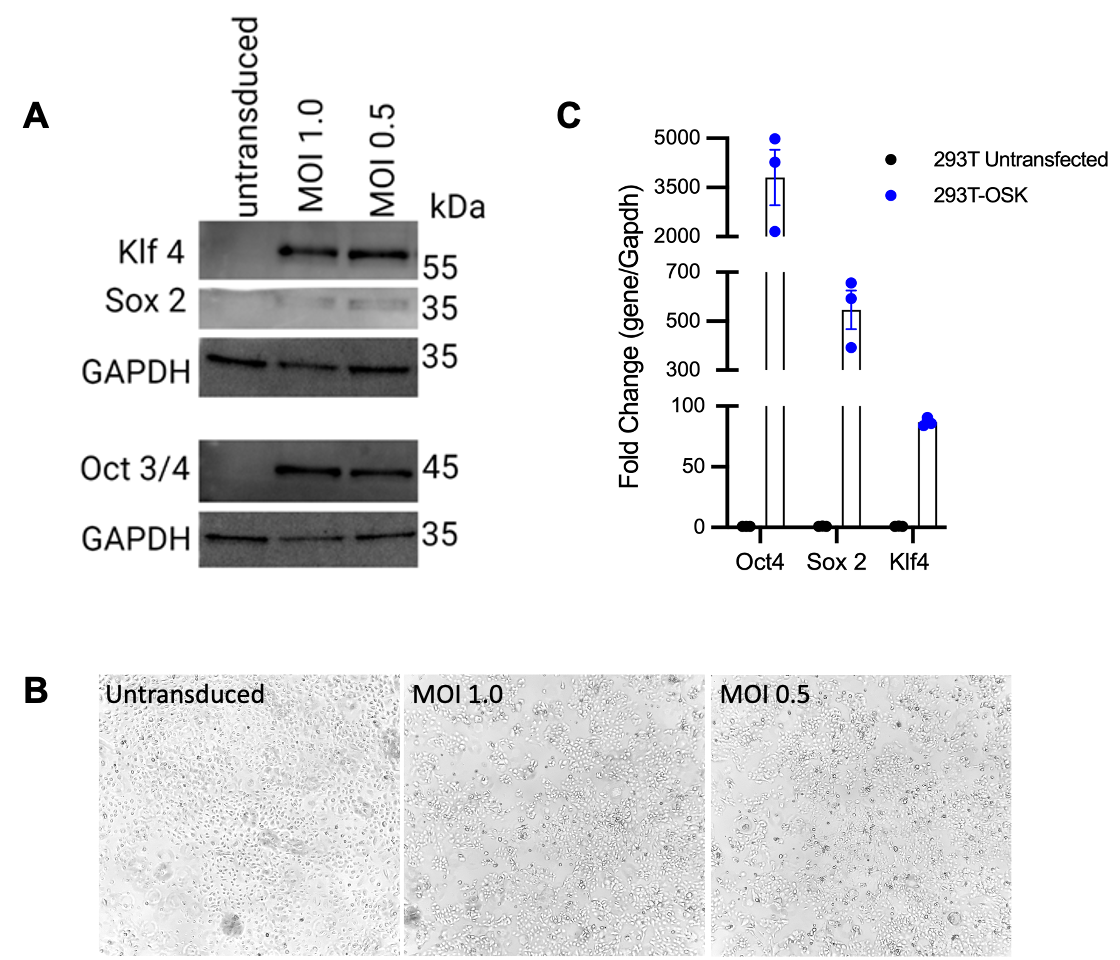


**Supplementary Fig. 3: Charactarization of in vitro lenti-OSK system.**

a. Immunoblots for OSK expression and b. bright field images of keratinocytes, at D21 post lentiviral transduction, two days before DNA collection; c, OSK mRNA level from lentiviral plasmid transfection in 293T cells.
